# Modelling the interrelationships between potential risk factors and childhood Co-morbidity of Malaria, Anaemia, and stunting in children less than five years in Burundi

**DOI:** 10.1016/j.heliyon.2024.e38525

**Published:** 2024-09-26

**Authors:** Rugiranka Tony Gaston, Shaun Ramroop, Faustin Habyarimana

**Affiliations:** School of Mathematics, Statistics and Computer Sciences, University of KwaZulu-Natal, Pietermaritzburg Campus, Private Bag X01, Scottsville, 3209, South Africa

**Keywords:** Malaria, Anaemia, Stunting, Interrelationships, SEM, And children younger than five years in Burundi

## Abstract

**Background:**

Anaemia, malaria, and stunting remain health problems, especially in children younger than five years, and those conditions are linked to morbidity and mortality. The main objective was to assess the relationships between anaemia, malaria, and stunting. Also, the current study aimed to understand the complex interrelationships between explanatory factors, and their direct or indirect relationship with childhood malaria, anaemia, and stunting in Burundi.

**Methods:**

The study used secondary data from the Demographic and Health Survey in Burundi (BDHS) conducted on the March 7, 2017, with a weighted sample size of 13611 children younger than five years. A multivariate structural equation model (SEM) was used to evaluate the interrelationships between dependent variables and their direct or indirect relationship with childhood malaria, anaemia, and stunting. SEMs diverge from other techniques, as they look at the effects on hypothesised relationships from both direct and indirect perspectives (Takele et al., 2023) [1]. The variables with statistical significance were set at a p-value <0.05.

**Results:**

The findings from this study indicated an association between anaemia, malaria, and stunting (p < 0.001). The environmental and household factors were statistically significant (p < 0.038 and p < 0.001 respectively) and positively impacted childhood malaria, anaemia, and stunting. The results also indicated that the household factors were statistically significant (p < 0.001) predictors of childhood malaria, anaemia, and stunting. Furthermore, the findings from this study revealed that geophysical factors have a positive significant (p < 0.001) impact on childhood malaria, anaemia, and stunting via the mediating of the household factors. Contrastingly, with the environmental factors as a mediator, we observe a negative significant (p < 0.001) impact on childhood malaria, anaemia, and stunting. Lastly, the results showed that demographic factors had a negative significant (p = 0.004) effect on childhood anaemia, malaria, and stunting via the mediating of household factors.

**Conclusion:**

The findings from this study revealed an association between malaria, anaemia, and stunting, which imply that these conditions could contribute to collaborative improvements in child well-being. In addition, child demographic, household, environmental, and geographic factors were direct and indirect important drivers of childhood malaria, anaemia, and stunting. Therefore, improving sanitation, access to clean water, nutrition practices, and health care, especially for children from rural areas, and uneducated mothers with poor backgrounds could help to control and eliminate stunting, anemia, and malaria in children younger than five years in Burundi.

## 1 Introduction

Childhood malaria, anaemia, and stunting remain major health problems worldwide, particularly in children younger than five years in developing countries [[1], [2], [3]]. Malaria, anaemia, and stunting are intersecting and associated with morbidity and mortality, with pregnant women and children younger than five years being the most susceptible [[4], [5], [6]]. Anaemia, malaria, and stunting can contribute to children's poor cognitive development, mental health issues, low academic achievement, and susceptibility to diseases. These effects can last until adulthood [[7], [8], [9]].

Malaria is transmitted to individuals through the bites of infected Anopheles mosquitos. Malaria parasites attack red blood cells, decreasing the number of blood cells in the body, which can lead to severe anaemia [3]. Malaria vector breeding and development increase during the rainy and humid seasons accompanied by high temperatures in areas of low altitude [10,11].

Anaemia is defined as a low hemoglobin level in the blood (Hb). In children younger than five years, anaemia is categorized as mild when the Hb is between 10.0 and 10.9 g/dl, moderate, if the Hb is between 7.0 and 9.9 g/dl, severe, for Hb < 7.0 g/dl, and normal if Hb ≥ 11.0 g/dl [[12], [13], [14]]. Iron deficiency is assumed to be the greatest cause of anaemia in roughly 50 % of cases. However, inadequate levels of folate, vitamin B12, protein deficiencies, nutrients, and several illnesses including malaria and diarrhea among others, can increase the risk of anaemia [15,16].

Stunting is long-term or chronic malnutrition, caused by lacking nutrients and other minerals [8]. The nutrition of a child can be estimated using various anthropometric indices in relation to the WHO growth standards [17,18]. The first one is stunting (height-for-age) which implies long-term or chronic malnutrition; wasting (low-weight-for-height) indicates acute malnutrition; and underweight (weight-for-age) which can be either stunting, wasting, or both [[17], [18], [19]]. The nutrition status is calculated using the Z-score for height-for-age (HAZ), weight-for-height (WHZ), and weight-for-age (WAZ). Children younger than five years are treated for stunting, wasting, or being underweight if their HAZ, WAZ, or WHZ are less than minus two standard deviations (<-2 SD) respectively. Moreover, children with Z-scores of less than three standard deviations (<-3 SD) were classified as having severe stunting, wasting, and/or being underweight respectively [14,17,18,20]. The Z-scores are normally obtained from the child's weight, height, or length and calculated as indicated in the following formula:

$z=\frac{x-\mu }{\sigma }$, where μ,andσ are the mean and standard deviation of the scores, while x is the values of length, weight, or height [20,21].

In 2019, the prevalence of anaemia in children younger than five years was 39.8 % globally, with the highest percentage (60.2 %) from Africa [8,14]. In 2017, the prevalence of malaria and stunting in children younger than five years globally was at 61 % and 26.6 % respectively. The highest percentages are from Africa with 92 % of malaria and 22.2 % of stunting cases [17,22]. In Burundi, the prevalence of malaria in children less than five years was 27 % and that of anaemia and stunting were 61 % and 56 % respectively in 2017 [23,24]. These percentages show that malaria, anaemia, and stunting are still health problems in children globally, especially in developing countries, and this includes Burundi. Addressing the anaemia, malaria, and nutritional conditions of children is essential to ensuring a high standard of living for children, especially those younger than five years [16].

Although efforts have been undertaken to control malaria, anaemia, and stunting through humanitarian and government interventions, the percentages show that malaria, anaemia, and stunting are still health problems in children.

The current study used a structural equation model (SEM) to understand the complex interrelationships between dependent variables and their direct or indirect association with childhood malaria, anaemia, and stunting in Burundi. The indirect effect modeling in the SEM allows the inclusion of alternative variables that may not be significant in direct effect models [25]. The direct effects are typically the relationships between the independent and dependent variables without any intermediate variable. However, the indirect effects are the relationships between the independent and dependent variables through a mediated variable [[26], [27]]. The direct and indirect effects can have the same signs or different signs. When the direct and indirect effects have a positive influence, means that there is a complementary mediation. In the case of direct or indirect effects having dissimilarity influence, there is a competitive mediation [27].

Structural equation modelling has become a general method in science for analysing and understanding multivariate relationships among the variables of interest. The analysis of covariance (interaction) using structural equations, also known as latent variable analysis, is a new area of statistics. However, this method has been applied in econometrics and psychometrics for a long time [[28], [29], [30]]. The SEM is usually a multivariate model that links an attribute and unmeasured latent variables [[31], [32], [33]]. The structural equation model can assess complex interrelationships between different variables and related unobserved and observed variables. The assessment can be done by calculating the sample covariance matrix of the observed variables and the population covariance matrix produced by the SEM framework [32,[34], [35], [36], [37], [38]]. The SEM is very important in its extension for calculating measurement errors via latent variables. Structural equation modelling allows the evaluation of numerous sets of observed variables to define the non-measurable variable (latent or construct variables) and allows these latent variables to be related to each other [39,40]. The SEM integrates several variables, which are not measured directly but along their effects or indicators. Furthermore, the SEM methodology involves multivariate data analysis tools that merge features of multiple regression and factor analysis. This allows for the simultaneous calculation of a series of interrelationships between variables and relations of dependency that permit the methodology of directly including a measurement error in the model [37,41,42]. The theoretical model of SEM can be demonstrated by applying mathematical equations and graphs (path diagrams) to summarise a set of hypotheses [33,39]. Moreover, SEM methodology can accommodate both observed and unobserved (latent or constructed) variables, which is one of the most important distinctions between structural equation modelling and other statistical modelling tools [25,37,43].

In the literature, most studies assessed the association between either anaemia and malaria; anaemia and stunting, or malaria and malnutrition using different statistical methods [3,18,28,[29], [44], [45]]. Few studies have tried to evaluate the association between anaemia, malaria, and malnutrition [5,14,30]. However, not all of these studies assessed the association between anaemia, malaria, and stunting simultaneously.

Hence, understanding the relationship between anaemia, malaria, and stunting, as well as other associated risk factors, makes it simpler to control and eradicate the prevalence of anaemia, malaria, and stunting. In addition, it aids in the design of intervention measures by various contributors and policymakers.

Thus, the current study used a structural equation model (SEM) to understand the complex interrelationships between dependent variables and their direct or indirect association with childhood malaria, anaemia, and stunting in Burundi. Furthermore, the study aims to identify if there are associations between anaemia, malaria, and stunting.

## 2 Methodology and material

### 2.1 Study area

Burundi is a small (27,834 km2) land-locked and densely populated country located in East Africa with Bujumbura as the capital city [46,47]. Burundi is one of the world's least poor countries, accounting for more than 70 % of the population living in poverty. The economy of the country is based on agriculture, where the majority of the population is employed in the agriculture sector [47,48]. Burundi is bordered by Rwanda to the north, Tanzania to the east and south, Lake Tanganyika to the southwest, and the Democratic Republic of the Congo to the west [46,47,49]. Malaria, anaemia, and stunting are the leading cause of mortality rate in the population, especially, in children younger than five years in the country.

### 2.2 Data source

This study used secondary data from the Demographic and Health Survey in Burundi (BDHS) conducted on March 7, 2017. The survey was organized by the Institute of Statistics and Economic Studies of Burundi (ISTEEBU) in collaboration with the 10.13039/501100004397Ministry of Public Health with the aim of the fight against AIDS. Funding was provided by the 10.13039/501100003442Government of Burundi, the 10.13039/100000200United States Agency for International Development (USAID), United Fund for Childhood (10.13039/100006641UNICEF), the 10.13039/100004423World Health Organization (10.13039/100004423WHO), The Swiss 10.13039/100019767Cooperation, and the Belgian 10.13039/100019767Cooperation. The ICF gave specialized help to the whole task as a feature of the DHS Program, which was subsidized by USAID, and whose goal is to offer help and technical assistance globally for the realization of population and health surveys.

### 2.3 Data sampling and design

All Women aged 15–49 years and children from 6 to 59 months who remained in or visited the chosen households the night before the survey were included in the study. The 2017 survey was a national representative of rural and urban areas, Bujumbura-Mairie, and other provinces in the country. The 2017 sample was drawn based on a stratified, two-stage area survey [46]. The first stage of sampling includes a selection of 554 Primary Sampling Units (PSU) or clusters from the enumeration areas (EAs) established during the General Population and Housing Census (GPHC) carried out in 2008. The second stage of sampling involved systematic selection with equal probability and included a sample of 30 households in each cluster from urban and rural areas. A total number of 16620 households were selected and 3180 were from urban areas in 106 clusters, while 13440 were from rural areas in 448 clusters.

### 2.4 Data weighting

The current study used a weighted sample of 13611 children aged between 6 and 59 months [50].

The weighted sample was used in the study to make inferences, which were representative of the country, and to account for the data set's complex sampling procedure. Individuals surveyed in each region should contribute proportionally to the size of the region's total sample in the sampling procedure. In any case, some regions may have small populations, and this unweighted provision may not accurately represent the population. As a result, the region with a small population is oversampled to tackle these challenges, and thus the weighted sample is used [3,46].

### 2.5 Study criteria and blood sampling methods

The inclusion criteria for this study were children aged between 6 and 59 months, who remained in or visited the selected households the night before the survey and were guided by their parents or guardians. While the exclusion criteria were children aged less than 6 months, and their parents or guardians were not able to communicate in Kirundi, French, or English [46]. The exclusion and system missing values were regarded as missing data and were removed.

With the parent's or guardian's permission, all children aged between 6 and 59 months were tested for anaemia, malaria, and stunting. The trained laboratory technicians and nurses conducted the process of testing for anaemia, malaria, and stunting. The blood sample for anaemia was collected using a spring-loaded, sterile lancet to make a finger- or heel-prick. The drop of blood was collected in a microcuvette, and the Haemoglobin level was analysed using a portable HemoCue analyzer. The blood sampling for malaria was collected from children's finger- or heel-prick using the SD Biolne Malaria Ag P.f/P, a rapid diagnostic test (RDT). We used a rapid diagnostic test (RDT) as is easy to access and learn, however, microscopy can also be used for malaria testing [11,46].

Lastly, the height measurement was taken using a tape board, where children under two years of age were measured in a lying position, while the older children were measured in a standing position. The weight measurement was taken using electronic scales (SECA) provided by UNICEF. The scale was aligned to zero and parents or guardians were asked to unclothe their children or keep them in light clothing. For the children who were unable to stand, the weight was calculated based on the difference in parent weight compared to the weight of the parent holding the child. Based on the children's weight, height, and age it was easy to calculate their nutritional status (i.e. Weight-for-age, height-for-age, and weight-for-height) based on the WHO guidelines [18,46].

All results were given to the child's parent or guardian in oral and composed structure and were recorded on the Biomarker Questionnaire. Moreover, children who tested positive for malaria, anaemia, or stunting were provided with a full course of medication [46].

### Ethics approval

The ethical approval for the 2017 BDHS was organised and assessed by the Institute of Statistics and Economics Studies of Burundi and approved by the Minister of Public Health.

## 3 Data analysis

### 3.1 Dependent variable

The dependent (response) variables of interest are malaria, anaemia, and stunting in children younger than five years in Burundi. In this study, a child was considered to have malaria if he/she tested positive or not infected if he/she tested negative using the RDT results. A child can be considered to have either severe (Hb < 7 g/dl), moderate (Hb between 7 and 8.9 g/dl), or mild (Hb between 9 and 10.9 g/dl) anaemia. However, in the current study, the response variable used was binary; hence, the child was considered anaemic only if the Hb level was below the altitude-adjusted threshold of 11 g/dl, otherwise. Lastly, the nutrition status of a child was also binary, and a child was considered malnourished (stunted) when the standard deviation (SD) was less than minus two standard deviations, otherwise, a child is normal (not stunted).

### 3.2 Independent variables

The independent (exploratory) variables used in this study were also used in previous studies on childhood anaemia, malaria, or malnutrition [18,51]. As a result, this provides the conceptual framework for the current study. These independent variables include demographic, household (socioeconomic), geographical, and environmental factors. The household factors include residence, wealth index, source of drinking water, mother's educational attainment and access to information through television, household access to electricity, type of toilet facility, the household share of toilet facility, as well as the household's main roof, floor, and wall material. The environmental factors include rainfall, proximity to water, land surface temperature, enhanced vegetation index (EVI), aridity, wet days, and clusters. The child's demographic factors include age, gender, and whether or not the child slept under a mosquito net. Finally, the geophysical factors include the geographical regions of the children, travel times, and nightlight composites.

## 4 Statistical analysis

### 4.1 Model formulation

The application of structural equation modelling includes various steps such as the development of the theoretical conceptual model; specification of the mathematical model; determination of the model's evidence; and determining the model fit and evaluation of the goodness-of-fit of the model [35,40].

#### 4.1.1 Theoretical conceptual and path models

The latent (constructs) variables cannot be measured directly and are known as theoretical concepts. The latent variable is measured by observed (indicator) variables, and they assist the expansion and estimation of casual relationships in SEM [35,52]. The latent variables related to each other in the model must be indicated first, and the impact that these variables apply to each other is categorized as exogenous and endogenous [39,40].

**Fig. 1:**
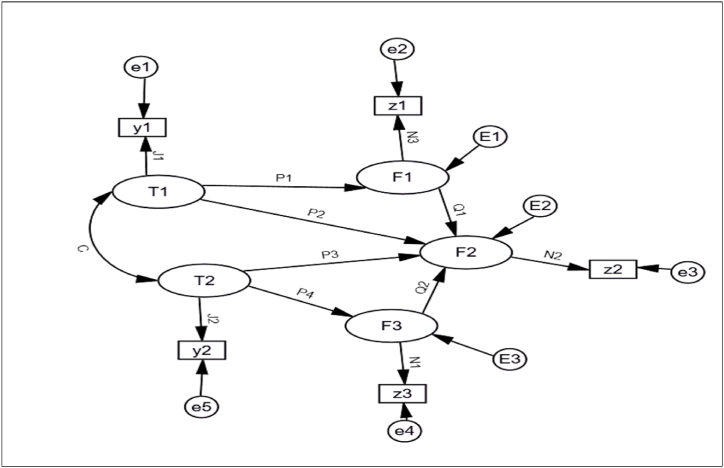
Graphical path of a structural model.

In the model $F_{i}$ are the latent endogenous variables; $T_{i}$ the latent exogenous variables; $z_{i}$ are the observed endogenous variables; $y_{i}$ the observed exogenous variables; $e_{i}$ is the measurement errors, $E_{i}$ are structural errors; c is the coefficient correlation between the latent exogenous variable, while $P_{i}$, $Q_{i}$, $J_{i}$, and $N_{i}$ are the coefficients as indicated in Fig. 1.

The exogenous (predictive) variables are not manipulated by the effect of other variables in the model and are measured without error. However, the endogenous (dependent) variables are influenced by the effect of other variables included in the model [43].

Structural equation modelling offers the unique capacity to describe latent or unobserved variables in a linear model, contrary to other statistical approaches where observed variables in a specific data set are employed for statistical analysis [34,35,53,54].

The SEM includes the measurement and structural models which can be written as follows:

(4.1) φ=αφ+τδ+ϵ,

where φ denotes a vector m×1 of latent endogenous variables, δ denotes a vector of k×1 latent endogenous variables, α represents an m×m matrix of coefficients connecting the latent endogenous variables to each order, τ denotes an m×k coefficients of the matrix that links the endogenous variables to the exogenous variables, and ϵ denotes an m×1 vector of the structural disturbances or errors. The main diagonal components of α are often zeros with δandϵ viewed as mutually independent and normally distributed [39,43,55]. The measurement model, which is theoretically described independently for the endogenous and the exogenous variables, linking the observable and latent variables together is expressed by:

(4.2) $$Y=\pi_{y}\varphi +\mu ,\text{and}\;X=\pi_{x}\delta +\theta \text{,}$$

Where $\pi_{y}$ (*p×m*) and $\pi_{x}$ (*q×k*) are coefficient matrices illustrating the relationship between latent endogenous and exogenous factors and the observable variables, respectively. Consequently, μandθ are *p×1* and *q×1* vectors of measurement errors in *Y* and *X* respectively.

In order to generate a scale for the related latent variables, each column of the π matrices normally has a value that is set to one. As an alternative, this can also be accomplished by setting the variances of the latent exogenous variables in a matrix defined as γ, and the matrix corresponding to the exogenous variable's covariance matrix to zero. Furthermore, the measurement model's fit to the confirmatory factor analysis (CFA) application, which identifies the latent components, must be addressed [35,39,40].

The CFA examines the theoretical measurement model and determines whether the hypothesised measurement model produces a variance-covariance matrix that is identical to the sample variance-covariance matrix. Based on equation (4.2), the measurement errors μ and θ, each having a multivariate normal distribution, are considered to have zero expectations.

The errors presume independent of each other, independent of latent endogenous variables φ, latent exogenous variables δ, and independent of the disturbances ϵ. Additionally, it is assumed that the latent exogenous variables have a multivariate normal distribution and that the observations are independently sampled. However, for the endogenous variables that are accurately measured, this assumption is irrelevant [39,40].

The structural errors ϵ, on the other hand, are unaffected by the latent exogenous variables δ and have zero expectation with a multivariate normal distribution. The observed indicators *X* and *Y* in this case exhibit a multivariate normal distribution and can be expressed as follows:

(4.3) $$\left(\begin{array}{c} X\\ Y \end{array}\right)\;᷉N_{p+q}\left(0,\rho \right)\text{,}$$

where ρ, is the indicators' population covariance matrix, which is a function of the model's parameter $\phi =\alpha ,\tau ,\pi_{x},\pi_{y},\eta ,\lambda_{\delta },{\pi \lambda }_{\epsilon },\beta$, ξ and can be calculated as:

(4.5) $$\rho =\left(\begin{array}{cc} \rho_{xx} & \rho_{xy}\\ \rho_{yx} & \rho_{yy} \end{array}\right)=\left(\begin{array}{cc} \pi_{y}{\left(1-\alpha \right)}^{-1}\left({\tau \gamma \tau }^{\prime }\right){\left[{\left(1-\alpha \right)}^{-1}\right]}^{\prime }\pi_{y}^{\prime }+\lambda_{\mu } & \;\pi_{y}{\left(1-\alpha \right)}^{-1}\tau \gamma \pi_{x}^{\prime }\\ \pi_{y}\gamma \tau^{\prime }{\left[{\left(1-\alpha \right)}^{-1}\right]}^{\prime }\pi_{y}^{\prime } & \pi_{x}\gamma \pi_{x}^{\prime }+\lambda_{\theta } \end{array}\right)\text{,}$$

where γ, indicates a *k×k* covariance matrix of the latent exogenous variables δ, τ denotes the *m×m* covariance matrix of the disturbance term, $\lambda_{\mu }$ and $\lambda_{\theta }$ represents the covariance matrices of the measurement errors *μ* and *θ*.

### 4.2 Model estimation

In order to obtain the matrix ρ, related to the confirmatory factor analysis (CFA) of Equation (1.5), it should be expected that α=0; τ=0;γ=0; $\pi_{y}=0$; and $\lambda_{\mu }=0$ [43]. In any given model, the restriction is essential in certain components of the matrix ρ, which incorporates setting a few parameters to zero. With adequate limitation, the maximum likelihood estimates (MLE) might be achieved for the parameters of the model and the log-likelihood related to the model can be portrayed as a function of the model's parameters of ρ and T, as the sampling covariance matrix between the observable variables. In the structural equation model, this method focuses on estimating the parameters ϕ, in order to minimise the inconsistency of function F(T,ρ). The inconsistency function F(T,ρ) is a scalar that estimates the distance between the examining covariance T and the adjusted covariance matrix $\hat{\rho }$ [53,56,57]. In structural equation modelling, the MLE and generalised least squares (GLS) are the most commonly used estimation methods [35,53,58]. The MLE methods are characterised by parameters so that the two matrices T and $\hat{\rho }$ are pretty much as close as could really be expected, for the likelihood logarithm estimates the vicinity between the two matrices. The asymptotic standard errors are calculated from the square root of the matrix diagonals, in the parameter estimates. It is also expected that the structural relationships between the latent endogenous variables φ and the latent exogenous variables δ are linear, as they are the interrelationships exuding between the indicator variables and the latent constructs.

### 4.3 Model identifiability

A principal step in model evaluation is to check the model's identifiability of latent variables, which is a complicated task in SEM without a straightforward answer [32,57]. The model can be reported as none identifiable when the system of equations cannot be solved. The counting rule for identifiability is generally the number of free parameters in the model, and must not be more than the number of variance and covariance between the observable variables, and can be shown as:

(4.6) $$\frac{\left(k+l\right)\left(k+l+1\right)}{2}\text{,}$$

where, k represents the number of endogenous variables and l represents the number of exogenous variables in the model [43,59].

However, the counting rule is not generally a sufficient condition, as the condition can be met yet still obtain a non-identifiable model. Hence, more conditions allow a model to be identified such as when measurement errors are not correlated when at least two exclusive indicators exist for each of the latent variables when a single indicator for latent variables is equally assumed without error is possible, and when the structural model includes only observed variables [35,40]. The SEM is not stable with a small sample size, and the minimum sample size depends on the involvement of the model, the degree of freedom, and the size effect [40,43].

### 4.4 Model diagnostics

In the structural equation model, the verification of the model fit is based on various goodness-of-fit-model criteria and is created to assess the model under several assumptions. In addition, verifying the goodness-of-fit is not a direct process as accessible in other multivariate techniques [39,43]. The chi-square ($\chi^{2})$ the test is widely regarded as the only statistical significance test commonly used to evaluate the theoretical model in SEM. The current study used a p-value less than 5 % as the cutoff for statistically significant variables.

The insignificant results indicate a resemblance between the original sample variance-covariance matrix and the variance-covariance matrix estimated by the model. Although, the application of $\chi^{2}$ is very difficult when the sample size is large, and rejecting the null hypothesis becomes hard [60,58]. When the chi-square has a zero value, it means there is a good fit or no difference between the values in the sample covariance matrix and the model-suggested covariance matrix (ρ) generated based on a theoretical model. However, in structural equation modelling, it is recommended that various goodness-of-fit criteria be used in conjunction with overall fit measurements [43,50,61]. As a result, the measurement indices range from poor fit to perfect fit, and various structural equation modelling programs report a range of the most common model fit as follows:

➢ Goodness of fit index (GIF) = 1-$\frac{\chi_{model}^{2}}{\chi_{null}^{2}}$ , where 1 indicates perfect fit
➢ Root mean square error of approximation (RMSEA) = $\sqrt{\frac{\chi_{model}^{2}-{df}_{model}}{\left\langle N-1\right\rangle {df}_{model}}}$, as a value less than 0.05 indicates the model's good fit.
➢ Comparative fit index (CFI) = 1-$\frac{\chi_{model}^{2}-{df}_{model}}{\chi_{null}^{2}-{df}_{null}}$, where the good fit the value greater than 0.9 is expected.
➢ Tucker-Lewis index (TLI) = $\left[\left(\frac{\chi_{model}^{2}}{\chi_{null}^{2}}\right)-\left(\frac{\chi_{model}^{2}-{df}_{model}}{\chi_{null}^{2}-{df}_{null}}\right)-1\right]$.
➢ Incremental fit index (IFI) = $\frac{\chi_{null}^{2}-\chi_{model}^{2}}{\chi_{null}^{2}-{df}_{model}}$, and
➢ Normal fit index (NFI) = $\frac{\chi_{null}^{2}-\chi_{model}^{2}}{\chi_{null}^{2}}$.

These mentioned above goodness-of-fit criteria are based on differences in variance-covariance matrices between observed (original, T) and model-implied (replicate, ρ) [43,53,57,59]. In addition, to check the validity and reliability of the internal consistency between various items, Cronbach's alpha (coefficient alpha) method was used in this study. Cronbach's alpha coefficient varies between 0 and 1, and the acceptable coefficient is ≥ 0.7 [[62], [63], [64]].

## 5 Model fit and data analysis

Initially, we evaluated a theoretical model for individual variables (household, environmental, child demography, and geophysical factors) to guarantee that the theoretical relationships between the observable variables and their corresponding factors were upheld by the data.

The CFA was used to evaluate whether the measurement model and relationships between all the latent and manifest variables are relevant. Numerous model fit indices were used in the analysis of this study, however, the common technique for assessing model fit is $\chi^{2}\text{,}$ and should not be significant for a good model [43,65]. Among the model fit indices and their conditions, the incorporated use of CFI, GFI, IFI, TLI, and NFI should be greater than 0.90 for a good model [53,57,61].

The RMSEA ≤0.05 indicates a best-fit model, however, the values between 0.05 and 0.08 show a reasonable fit model [50,59,61]. The validity of the structural model used a cross-validation method, which includes categorizing the data into two different sample sizes [40,59]. The first sample was considered as an adjustment sample, while the second was the validation sample. We first tested the SEM on the adjustment sample and analysed the goodness-of-fit model, once the model reached a good fit for the adjustment sample, the model could be assessed on the validation sample [40,50]. The validity of the model was obtained when the covariance structure of the model reached the best fit in both the adjustment and validation samples. The maximum likelihood estimation techniques are used for full structural equation modelling in the calibration sample. In general, the less value of $\chi^{2}\text{,}$ the better goodness of the fit to the data. However, we cannot depend only on ${\;\chi }^{2}$, as the test statistics are sensitive when the sample size is large and tend to reject the model [43,50,66]. Therefore, other indices, which are not depending on the sample size, are included. These indices and their cut-off indicating a good fit are NFI ≥0.95, CFI ≥0.95, AGFI ≥0.90, and RMSEA ≤0.07 [35,40,50,67].

In the theoretical model, we used the household factors (residence, wealth index, source of drinking water, type of toilet facility, the household share of toilet facility, mother's educational attainment, mother's access to information through television, household access to electricity, household's main roof, floor, and wall material); environmental factors (rainfall, proximity to water, land surface temperature, enhanced vegetation index (EVI), Aridity, wet days, and cluster); and child demographic factors (child's age, child's gender, and child slept under a mosquito net) to determine whether they directly or indirectly influence childhood malaria, anaemia, and stunting. The geophysical factors (geographical regions of the children, travel times, and nightlight composites) were determined whether they are directly or indirectly related to childhood malaria, anaemia, and stunting through the mediating effects of household factors.

Relying upon those assumptions, the conceptual framework can be defined by latent variables, which are deduced through observable variables because they cannot be directly measured, to assess their impact on childhood malaria, anaemia, and stunting co-morbidity. In a full SEM, the latent factors are then normalized on other factors based on the theory, empirical research, and suitable observed indicators [43].

**Fig. 2:**
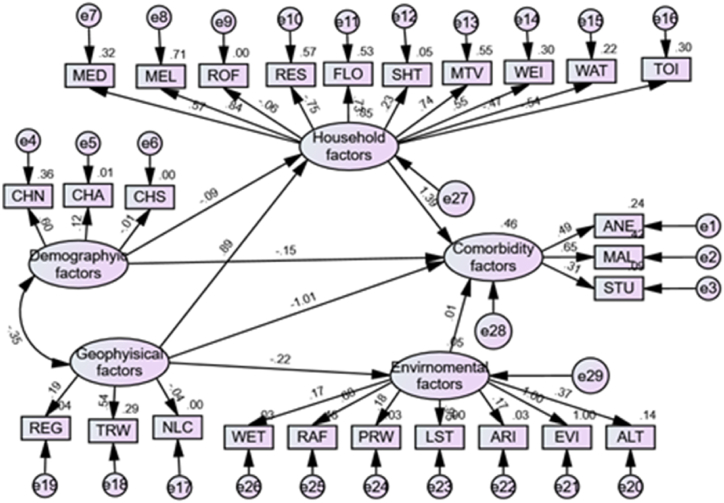
Theoretical full structural model.

In order to assess the model fit and evaluate the variables of interest (child demography, household-level, environmental, and geophysical factors), we used the two approaches of equations (4.1), (4.2) by conducting a confirmatory factor analysis (CFA) to test the hypothesised associations among these variables. We then created a conceptual path model diagram (Fig. 2) that comprised the endogenous and exogenous variables to represent the causal structure shown in the figure and define all of the conceptual relationships between these factors with respect to childhood co-morbidity. Thereafter, we evaluated the estimation model with all latent and observed factors included in the full model. The association between the direct and indirect variables, together with childhood malaria, anaemia, and stunting was assessed using structural techniques. For the acceptance or rejection of the model, we performed a goodness of fit test using SPSS AMOS software version 27.0 [66,68].

## 6 Results and interpretations

All exploratory variables were included in the model and were removed one by one to get the best-fit model. Fig. 2 indicates the results of the full model, after deleting some items in the model for the best fit. The household constructs had 11 factor higher order construct comprising: residence, wealth index, source of drinking water, type of toilet facility, the household share of toilet facility, mother's educational attainment, and mother's access to information through television, household access to electricity, household's main roof, floor, and wall material, the Cronbach's α score (0.84) was acceptable after deleting one variable (household's main wall material) from the model, and 10 items left as indicated in Fig. 2.

For the environmental factors, Cronbach's α score (0.79) was acceptable after deleting two variables (maximum and minimum temperature) from the model and left with seven. The geophysical factors resulted in a higher order of three factors in the model comprising geographical regions of the children, travel times, and nightlight composites; and Cronbach's α score (0.90) was acceptable. The child demographic factors (child's age, child gender, and child sleeping under mosquito net) also had an acceptable Cronbach's α score (0.81).

The non-significant Bollen-Stine p statistic, together with the underlying model statistics, demonstrated that the model was a good fit for the data in each testing model case, illustrating factor validity [59,67,68]. The Cronbach's α score in every case was above the recommended cut-off of 0.75. This shows an adaptable model, internal consistency, and good scale reliability between the constructs (items) in the model [59,27].

Then we improved the CFA model to get a better model with the highest goodness-of-fit as indicated in Table 1. The calibration sample model fit results showed that changing the model is not effective. The standardized regression weights for this model were all significant at a 5 % significant level. The full path results of SEM are indicated in Fig. 2.

**Table 1:** The goodness-of-fit indices in the two models (CFA and SEM).

MODEL	$\chi^{2}/df$	GFI	CFI	IFI	TLI	NFI	RMSEA
**Conceptual CFA model**	732.025	0.651	0.645	0.640	0.545	0.648	0.046
**Full structural model**	435.020	0.968	0.976	0.926	0.912	0.913	0.04

Both the direct and indirect effects of geophysical, and demographic factors are statistically significant (p < 0.001) on childhood malaria, anaemia, and stunting.

The direct and indirect interrelationships between contextual factors and their impact on childhood malaria, anaemia, and stunting are summarised in Table 2 and 3.

**Table 2:** Standardised direct and indirect effects of factors on childhood malaria, anaemia, and stunting. Note: the ∗ on the numbers indicates the p-values. p∗≤ 0.05; p∗∗≤ 0.01, and p∗∗∗≤ 0.001.

Factors	Total effect	Direct effect	Indirect effect
**Geophysical factors → Household**	0.889∗	0.889∗	–
**Geophysical factors → Environmental**	−0.217∗∗	−0.217∗∗	–
**Geophysical factors →Co-morbidity**	0.231∗	−1.006∗	1.237∗
**Household factor→Co-morbidity**	1.394∗	1.394∗	–
**Child demography factor→Co-morbidity**	−0.271∗	−0.150∗	−0.121∗
**Child demography factor→Household**	−0.087∗∗∗	−0.087∗∗∗	–
**Environmental → Co-morbidity**	0.014∗∗	0.014∗∗	–

**Table 3:** Standardised regression coefficients for the full structured model.

	Parameters	Estimate	95%CI Lower	95%CI Upper	P-value
**Environmental factors**	Geophysical factors	−0.217	0.802	0.908	<0.001
**Household factors**	Demographic factors	−0.087	0.881	0.956	0.004
**Household factors**	Geophysical factors	0.889	2.305	2.573	<0.001
**Comorbidity factors**	Environmental factors	0.014	0.967	1.062	0.038
**Comorbidity factors**	Household factors	1.394	3.193	5.150	<0.001
**Comorbidity factors**	Demographic factors	−0.150	0.820	0.904	0.010
**Comorbidity factors**	Geophysical factors	1.006	0.286	0.461	<0.001
**ANE**	Comorbidity factors	0.493	1.481	1.809	<0.001
**MAL**	Comorbidity factors	0.650	1.691	2.164	<0.001
**STU**	Comorbidity factors	0.306	1.256	1.468	<0.001
**FLO**	Household factors	0.731	1.958	2.203	<0.001
**WAT**	Household factors	−0.465	0.608	0.649	<0.001
**MEL**	Household factors	0.842	2.259	2.385	<0.001
**ROF**	Household factors	−0.062	0.929	0.951	<0.001
**TOI**	Household factors	−0.544	0.563	0.599	<0.001
**WEI**	Household factors	0.549	1.649	1.818	<0.001
**MTV**	Household factors	0.739	2.052	2.136	<0.001
**SHT**	Household factors	0.230	1.246	1.271	<0.001
**RES**	Household factors	−0.752	0.458	0.485	<0.001
**MED**	Household factors	0.567	1.659	1.874	<0.001
**ALT**	Environmental factors	0.371	0.916	2.293	<0.001
**EVI**	Environmental factors	0.999	1.442	5.114	<0.001
**ARI**	Environmental factors	0.171	0.709	1.986	<0.001
**LST**	Environmental factors	0.951	1.598	4.191	<0.001
**PRW**	Environmental factors	0.178	0.685	2.085	<0.001
**RAF**	Environmental factors	0.680	1.239	3.146	<0.001
**WET**	Environmental factors	0.170	0.725	1.939	<0.001
**REG**	Geophysical factors	0.194	1.096	1.344	<0.001
**TRW**	Geophysical factors	0.535	1.099	2.654	<0.001
**NLC**	Geophysical factors	−0.036	0.603	1.543	<0.001
**CHN**	Demographic factors	0.603	1.702	1.962	<0.001
**CHS**	Demographic factors	−0.007	0.963	1.024	0.043
**CHA**	Demographic factors	0.120	1.033	1.231	<0.001

***ANE****: Anaemia,****MAL****: Malaria,****STU****: Stunting****, FLO****: Type of floor material,****WAT****: Type of drinking water,****MEL****: Mother's access to electricity,****ROF****: Type of roof material,****TOI****: Household has toilet facility,****WEI:****Wealth index,****MTV****: Mother's access to television,****SHT****: Whether household share toilet facility,****RES****: Type of place of residence,****MED****: Mother's education,****ALT****: Cluster altitude****, EVI****: Enhanced vegetation index,****ARI:****Aridity,****LST:****Land surface temperature,****PRW****: Proximity to water,****RAF:****Rainfall,****WET****: Wet days,****REG****: Region,****TRW:****Travel times,****NLC****: Night light composite,****CHN****: Child slept under a mosquito net****, CHS:****Child's sex,****CHA****: Child's age*.

In this study, the testing for the partial mediation effect assumed the estimation of two phases. The direct effect was used in phase one to estimate the effect of predictors (household, demographic, environmental, and geophysical factors) on childhood malaria, anaemia, and stunting. The direct path coefficient from both geophysical and demographic factors on malaria, anaemia, and stunting was statistically significant (p = 0.001) and revealed a negative direct effect on childhood malaria, anaemia, and stunting as indicated in Table 2. The negative sign shows the opposite directions between dependent and independent variables. The possible reason might be increasing the number of children sleeping under a mosquito bed net in the malaria region can reduce malaria, anaemia, and stunting. Furthermore, as children grow the chances of having malaria, anaemia, and stunting reduce [[3], [4], [5]].

However, the direct path coefficient from both household and environmental factors on malaria, anaemia, and stunting was statistically significant (p = 0.004) and revealed a positive direct effect on childhood malaria, anaemia, and stunting. The previous studies showed that household and environmental factors used in this study could have a positive impact on childhood malaria, anaemia, and stunting [17,29,49]. This is due to some variables from household and environmental factors presented in this study such as residence area, wealth index, altitude, toilet, and water facilities contribute positively to childhood anaemia, malaria, and stunting [3,17,29,49].

There was a negative direct association between geophysical and environmental factors (β = −0.217, p = 0.008). The geophysical factors (regions of a child, travel times, and nightlight composites.) are assumed to be related to environmental factors [68,69].

We also observed a negative direct association between child demographic and household factors (β = −0.087, p < 0.001). This might be because demographic factors (child age, gender, and child sleeping under mosquito net) are from household factors [50]. Furthermore, we observed a positive interrelationship between geophysical and child demographic factors.

In the second phase, we involved the testing the indirect relationship between geophysical and demographic factors on childhood anaemia, malaria, and stunting.

The geophysical factors had a positive indirect association with childhood malaria, anaemia, and stunting via the mediating effect of the household factors. The positive indirect effect it might be addressed by the socio-economic factors in the household. This is confirmed by previous studies [1,70,71], indicating that improving socio-economic, results in a reduction of childhood anaemia, malaria, and stunting.

Contrastingly, this indirect association was negative with the mediating effect of environmental factors. Environmental factors such as low altitude (<2400 m) and high (≥2400 m) temperature in previous studies have shown a negative effect on childhood malaria, anaemia, or malnutrition [2,10,29,72]. This is because childhood anaemia, malaria, and stunting are high in children living in low-altitude areas [2].

The demographic factors had a negative indirect association with childhood naemia, malaria, and stunting via the mediating effect of household factors. In this study, we used age and children sleeping under mosquito bed nets as demographic factors and these variables are from household factors. As child age increases, children are not likely to sleep under a mosquito bed net, and this increases childhood malaria, anaemia, and stunting. These results are in line with the previous studies [2,15,71,73].

The present study also indicated a positive association between malaria (0.65), anaemia (0.49), and stunting (0.31) (p < 0.001). This means that any change in either one or two disease(s) has a positive impact on the other (s). The findings from the current study are in line with the previous studies [3,18,74,75].

## 7 Strength and limitations

Structural Equation Model (SEM) was good to fit the data and indicated the complex interrelationships between geographical, child demography, household, and environmental factors, as well as their direct or indirect relationship with childhood anaemia, malaria, and stunting in Burundi. In addition, the sample size used in the current study is quite large.

However, SEM could not address the trends of the association between the predictors and response variables if they were linearly or nonlinearly associated. The present study also did not consider wasting and being underweight as a measurement of nutrition status. Lastly, the dataset was cross-sectional and could not study the change in factors and prevalence over time. Hence, a further study should be conducted to address these issues.

## 8 Summary and conclusion

The present study used the structural equation model, to demonstrate the complex interrelationships between the variables of interest and fitted our data well. We assumed that the observed variables are dependent on the latent variables. The model fits our data well and explains the complex interrelationships between variables in a dataset. The findings from this study indicate an association between anaemia, malaria, and stunting. In addition, results from the current study revealed that the geographical factors were statistically significant determinants of childhood malaria, anaemia, and stunting, and have a direct and indirect effect on childhood anaemia, malaria, and stunting. The estimated indirect path for the effect of geophysical factors on childhood anaemia, malaria, and stunting, as mediated by household factors was statistically significant and positive.

However, the estimated indirect paths for the effect of geophysical factors on childhood anaemia, malaria, and stunting factors, as mediated by environmental factors were statistically significant but negative. The child demography factors such as the child's age, child's gender, and child sleeping under a mosquito net, were statistically significant predictors of childhood anaemia, malaria, and stunting. The estimated indirect path effect on childhood anaemia, malaria, and stunting via the mediating effect of household factors was statistically significant and negative.

The household factors comprising residence, wealth index, source of drinking water, type of toilet facility, the household share of toilet facility, mother's educational attainment, mother's access to information through television, the household has access to electricity, household's main roof, floor, and wall material were also statistically significant predictors in childhood anaemia, malaria, and stunting. The study also indicates that environmental factors such as rainfall, proximity to water, land surface temperature, enhanced vegetation index, Aridity, wet days, and cluster were statistically significant predictors of childhood anaemia, malaria, and stunting. The findings from this study will assist Burundian policymakers and healthcare professionals in developing protective measures and planning mediation systems that target children younger than five years.

In order to develop effective intervention strategies to help reduce anaemia, malaria, and stunting in children younger than five years. The authors recommended that the Burundian government and policymakers should focus on fighting against anaemia, malaria, and stunting in children younger than five years. To achieve this, each household should be educated on measures and prevention of anaemia, malaria, and stunting, through social media, and workshops. In addition, the healthcare, toilet facilities, sleeping under mosquito bed nets, and use of clean water should be improved; especially, for individuals from rural areas, uneducated mothers, and poorer quantile index households. Furthermore, improving the nutritional status of children mostly from rural areas, uneducated mothers, and poorer quantile index households will reduce anaemia, malaria, and stunting in children younger than five years.

## Funding statement

The authors declare that there was no funding associated with this study.

## Institutional review board Statement

Not applicable.

## Additional information

No additional information is available for this paper.

## Data availability statement

The dataset used during this study can be accessed for free after registration on the DHS programme using the following link:

https://www.dhsprogram.com/data/dataset_admin/login_main.cfm.

## CRediT authorship contribution statement

**Rugiranka Tony Gaston:** Writing – review & editing, Writing – original draft, Visualization, Validation, Software, Methodology, Investigation, Formal analysis, Data curation, Conceptualization. **Shaun Ramroop:** Validation, editing, Project administration, Resources, Supervision. **Faustin Habyarimana:** Validation, editing, Project administration, Resources, Supervision.

## Declaration of competing interest

The authors declare that they have no known competing financial interests or personal relationships that could have appeared to influence the work reported in this paper.
